# Multilayer Plasmonic Nanostructures for Improved Sensing Activities Using a FEM and Neurocomputing-Based Approach

**DOI:** 10.3390/s22197486

**Published:** 2022-10-02

**Authors:** Grazia Lo Sciuto, Christian Napoli, Paweł Kowol, Giacomo Capizzi, Rafał Brociek, Agata Wajda, Damian Słota

**Affiliations:** 1 Department of Electrical, Electronics and Informatics Engineering, University of Catania, Viale Andrea Doria, 6, 95125 Catania, Italy; 2 Department of Mechatronics, Silesian University of Technology, Akademicka 10a, 44-100 Gliwice, Poland; 3 Department of Computer, Control, and Management Engineering, Sapienza University of Rome, Via Ariosto 25, 00185 Rome, Italy; 4 Department of Mathematics Applications and Methods for Artificial Intelligence, Faculty of Applied Mathematics, Silesian University of Technology, 44-100 Gliwice, Poland; 5 Institute of Energy and Fuel Processing Technology, 41-803 Zabrze, Poland

**Keywords:** solar cell, surface plasmon polaritons (SPPs), cascade forward neural network (CFNN), finite element analysis (FEM)

## Abstract

In order to obtain optimized elementary devices (photovoltaic modules, power transistors for energy efficiency, high-efficiency sensors) it is necessary to increase the energy conversion efficiency of these devices. A very effective approach to achieving this goal is to increase the absorption of incident radiation. A promising strategy to increase this absorption is to use very thin regions of active material and trap photons near these surfaces. The most effective and cost-effective method of achieving such optical entrapment is the Raman scattering from excited nanoparticles at the plasmonic resonance. The field of plasmonics is the study of the exploitation of appropriate layers of metal nanoparticles to increase the intensity of radiation in the semiconductor by means of near-field effects produced by nanoparticles. In this paper, we focus on the use of metal nanoparticles as plasmonic nanosensors with extremely high sensitivity, even reaching single-molecule detection. The study conducted in this paper was used to optimize the performance of a prototype of a plasmonic photovoltaic cell made at the Institute for Microelectronics and Microsystems IMM of Catania, Italy. This prototype was based on a multilayer structure composed of the following layers: glass, AZO, metal and dielectric. In order to obtain good results, it is necessary to use geometries that orthogonalize the absorption of light, allowing better transport of the photocarriers—and therefore greater efficiency—or the use of less pure materials. For this reason, this study is focused on optimizing the geometries of these multilayer plasmonic structures. More specifically, in this paper, by means of a neurocomputing procedure and an electromagnetic fields analysis performed by the finite elements method (FEM), we established the relationship between the thicknesses of Aluminum-doped Zinc oxide (AZO), metal, dielectric and their main properties, characterizing the plasmonic propagation phenomena as the optimal wavelengths values at the main interfaces AZO/METAL and METAL/DIELECTRIC.

## 1. Introduction

Plasmonic nanostructures can be used to control and manipulate light in the visible and infrared spectrum, and have been utilized in combination with optoelectronic devices to selectively enhance light absorption or to increase the efficiency of light emission. Biosensing is one particularly important application area: the properties of localized plasmons or propagating surface plasmon polaritons (SPPs) are influenced by refractive index change in their vicinity, which enables the detection of ligand–analyte binding events [1].

Due to the complex fabrication technology, the optimized devices (photovoltaic modules, power transistors for energy efficiency, and high-efficiency sensors) often feature sophisticated stacks of several semiconductor and passivation layers with varying refractive indices and thicknesses. While those layers serve a practical purpose, e.g., as etch-stop layers during processing or for the integration of Ge photodetectors on Si substrates, their geometric parameters can also serve as optimization parameters for device performance as an integrated refractive index sensor. Layer thicknesses have been shown to influence the shape of the Fano resonance in a device in which an Al nanohole array is integrated into the metallization of Ge-on-Si photodetectors [1,2].

Metals have a proven history as materials used to fabricate plasmonic nanostructures/nanoparticles with remarkable properties, including enhancement in photothermal/photocatalytic activity, surface-enhanced Raman scattering (SERS), and metal-enhanced fluorescence (MEF). Among said applications, enhancement in MEF is an area of particular interest due to its wide-range usability in photonics, medical diagnostics, and nanobiotechnology. Variations in the type of materials, composition, and geometric design of nanostructures significantly affect the photodegradation resistance, fluorescence intensity, fluorophores photostability, and general sensitivity of devices based on plasmonic nanostructures [3].

The surface plasmon polaritons (SPPs) in basic terms are defined as electromegnetic waves that arise via the coupling of the conductor electrons oscillations with the electromagnetic fields. Their characteristics include a wide frequency range, propagation along metal–dielectric interfaces, and decay in the perpendicular direction. SPPs occur at the metal/dielectric interface during the coupling of a free metal electron to a photon. This phenomenon can be described as quantized charge density oscillations [4,5,6].

Enhancement of absorption efficiency in thin film structure can be achieved using plasmonics [7,8,9]. Therefore, it is possible to make micro–nano optoelectronic devices using the properties of the surface polarization wave, which is conducive to the realization in miniaturization of optoelectronic integrated devices. In recent decades, the research on surface plasmon polaritons relies on precious metal materials represented by gold and silver. Using these precious metal materials, researchers have proposed a large number of plasmonics structures [10,11,12,13] that can bind the light field at the nanometer level [14].

Thin-film second-generation silicon multilayer plasmonic structures provide a way to reduce production costs and improve the plasmonic structure sensitivity.

The implemented research allowed for the consideration of the most important properties of SPPs, as well as for the determination of their dispersion dependence on changes in the multilayer plasmonic structures. The variable here was the thicknesses of the different layers of the structure, namely glass, oxide, metal, and dielectric layers, while the excitation frequency was the constant.

The application of neural networks in plasmonics problems is not reported in the literature. The prediction of transmission lines is the reason for developing a new Artificial Neural Network (ANN)-based model, in which plasmonic and coupled nanobelts are simulated.

By using finite elements method (FEM) and ANN-based computing, the plasmonic phenomena investigation is proposed in terms of propagation characteristics in a multilayer structure with an embedded Aluminum-doped Zinc oxide (AZO). The latter is a promising indium-free TCO material for optoelectronics and photovoltaics applications, presented by Dr Salvatore Lombardo, Research Director of Italian National Research Council (CNR) at the Institute for Microelectronics and Microsystems IMM of Catania, Italy.

A factor that critically influences the efficiency of multilayer plasmonic structures is the nature and quality of the interface with the electrodes, one of which is typically constituted of transparent (semi)conductive oxide (TCO).

In particular, the TCO/metal interface contributes significantly to the internal resistance of the structure and is the site of degradation processes. Through the use of the Aluminium-doped zinc oxide (AZO) as TCO material and molibdenium as a metal, it is possible to minimize internal resistance and slow down the degradation processes [15,16,17,18].

AZO material can be described as a semiconductor with a wide range of conductivity, which also has the ability to change conductivity under different environmental conditions. Compared to other materials with similar properties, it is inexpensive, widely available, and exhibits non-toxicity. Advantages of AZO include its ability to achieve high transmission with good conductivity characteristics. Features that are somewhat limiting for the use of this material include, in particular, sensitivity to moisture [19,20,21].

Each set of thicknesses leads to a couple of values of $\lambda_{SPP}$ and $L_{SPP}$, the main quantities characterizing the SPP propagation, which will be defined by formulae in the next section.

The nanoplasmonic structure has been developed in COMSOL 3.5a RF Modul. The developed three-dimensional model simulates the electromagnetic field using the electromagnetic wave effect. The electromagnetic field is then calculated by COMSOL for various thicknesses of the AZO and Metal. For each couple of thicknesses, the values of $\lambda_{SPP}$ and $L_{SPP}$ are calculated by Matlab. A cascade forward neural network (CFFNN) has been used to explore the inner relationships between the exciting wavelength of SPPs, AZO, and Metal thicknesses and $\lambda_{SPP}$ and $L_{SPP}$.

The aim of this research is to achieve the optimal wavelengths of the SPPs to improve our understanding of plasmonic behavior at interfaces. The ability to predictably manipulate the associated electric fields of these plasmons has resulted in entirely new paradigms for chemical sensing. Significant efforts are being put forth in the overlapping areas of plasmonic nanostructures for surface-enhanced Raman spectroscopy (SERS), surface plasmon resonance (SPR) and metal-enhanced fluorescence (MEF)-based sensing, as well as the related fields of plasmonic energy harvesting and plasmonic device development. On the other hand, the theoretical calculations of $\lambda_{SPP}$ and $L_{SPP}$ refer to infinitely extended ideal plasmonic structures in which there are no defects in the materials. In addition, in these calculations, the permittivity of materials is considered constant when accompanied by the frequency of the incident wave change. Therefore, for the calculation of $\lambda_{SPP}$ and $L_{SPP}$ in real plasmonic structures, it is necessary to use the solution of Maxwell’s equations by means of the finite element method. However, FEM models are very computationally intensive, and such an approach is unfeasible when seeking the optimal construction parameters for an extended range.

The outcome of the proposed approach is a drastic reduction in execution times (from hours with the FEM solution to seconds with the neural network) for the model of a physical cell, which in turn makes it possible to find an optimal design for a plasmonic structure.

## 2. Basics of SPPs Propagation and Problem Formulation for a Multilayer Structure

**Figure 1:**
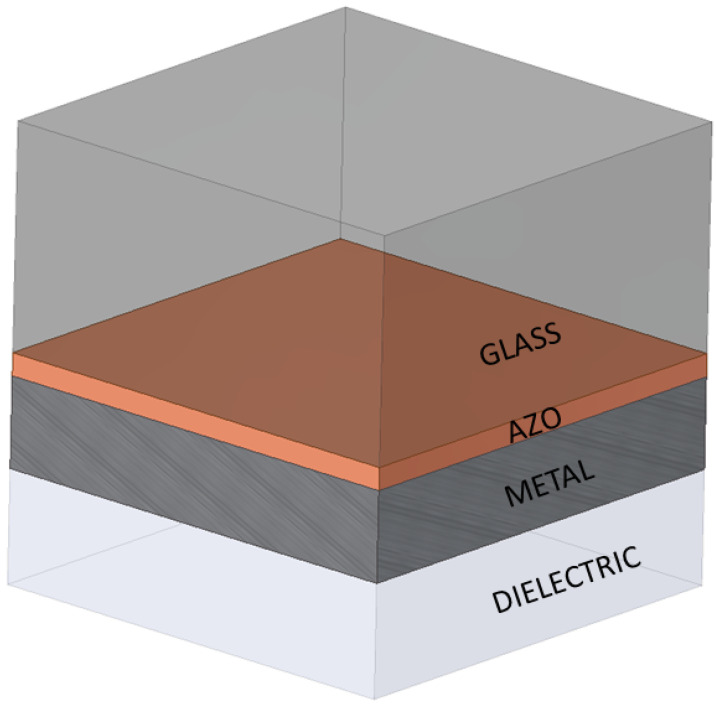
Structure of the thin-film solar device. The AZO (conductive aluminium-doped zinc oxide) layer is placed between the glass and the metal layers.

In studying the phenomena occurring at the metal/dielectric interface, the coupled electromagnetic wave SPP is an important excitation mode. The flat interface is the simplest geometry within which the SPP can be defined. In such a system, two half-spaces can be distinguished: a non-absorbing one with a real dielectric constant $\varepsilon_{2}$, whose value is positive, and a neighboring, conducting one defined by the dielectric function $\varepsilon_{1}\left(\omega \right)$. The effect of having to use a metallic material is the relation $Re[\varepsilon_{1}]<0$. This specific condition for metals is fulfilled for frequencies whose value is lower than the value of the plasmonic mass frequency $\omega_{p}$ [22]. The boundary conditions are determined for each medium present in the system. Acting on the basis of Maxwell’s equations, the SPP electromagnetic field is obtained. In this paper, a multilayer structure consisting of glass, AZO, metal, and dielectric layers is investigated. This structure is shown in Figure 1.

We use this structure because it reduces the computational effort for the investigations on the relation between dispersion and thickness of metal. However, this relation is unaffected by the complexity of the structure.

### 2.1. The Formulation of Propagation Phenomena for SPP

The boundary conditions are determined for each medium present in the system. Acting on the basis of Maxwell’s equations, the SPP electromagnetic field at contact surface is obtained. In the considered system, two types of materials can be distinguished: metal and AZO. The thickness of the dielectric is fixed at 1.44 μm, whereas the thicknesses of the layer of AZO and metal change in a specific range.

In order to introduce the main parameters characterising SPPs, assuming the interface is normal to z-axis and the SPPs propagate along the x direction (i.e., $k_{y}=0$), the SPP wavevector $k_{x}$ or β is related to the optical frequency ω through the dispersion relation.

(1) $$k_{x}=k_{0}\;\sqrt{\frac{\varepsilon_{d}\;\varepsilon_{m}}{\varepsilon_{d}+\varepsilon_{m}}}$$

(2) $$\beta =\frac{\omega }{c}\;\sqrt{\frac{\varepsilon_{d}\;\varepsilon_{m}}{\varepsilon_{d}+\varepsilon_{m}}}$$

The ω is considered real and $k_{x}$ is complex, since our main interest is in stationary monochromatic SPP fields in a finite area, where

(3) $$k_{0}=\frac{\omega }{c}$$

is the wavevector in free space, and $\lambda_{0}=\frac{c}{\omega }$ is the wavelength in a vacuum. For metals, the permittivity is complex, which leads to $k_{x}$ being complex.

The imaginary part of $k_{x}$ defines the SPP’s damping as it propagates along the surface.

The real part of $k_{x}$ is connected to the plasmon’s wavelength, $\lambda_{SPP}$:

(4) $$\lambda_{SPP}=\frac{2\pi }{Re[\beta ]}$$

Finally, the $L_{SPP}$ is defined as the SPP propagation length. Physically, the energy dissipates through the metal heating, which provides the propagation distance. $L_{SPP}$ is determined as follows:

(5) $$L_{SPP}=\frac{1}{Im[\beta ]}$$

However, our attention is focused on the solution of the electromagnetic fields in multilayer structure, and the relative alternating medium, as shown in Figure 1, and the concerning equations must be replicated. In this structure, each interface is involved in the plasmonic phenomena and sustains SPPs. Finally, the following equation provides the expression of the electric field of the plasmon wave:

(6) $$E_{SPP}=E_{0}^{\pm }\;e^{i(k_{x}x\pm k_{z}z-\omega t)}$$

where

$$\begin{array}{ccc} k_{x} & = & k_{x}^{{}^{\prime }}+ik_{x}^{\prime \prime }\\ k_{x}^{\prime } & = & \frac{2\pi }{\lambda_{SPP}} \end{array}$$

### 2.2. The Adopted Structure in the FEM-Based Simulations

Figure 1 shows the model of the multilayer structure used in the simulations. It includes the following layers: glass, AZO, metal, and dielectric. The domain dimensions were determined as values of 5.28 μm × 5.28 μm. The individual layers have specific thicknesses, with the glass layer (2.64 μm) and the dielectric layer (1.44 μm) being at a constant level. The thicknesses of the other two layers change within a fixed range. The external excitation is obtained by a probe located on top of structure obtaining an incident electromagnetic normally to the glass layer.

As a result, in the excitation of surface plasmon modes between the layers, here: at the AZO/metal interface and at the Metal/dielectric interface, the AZO film exhibits very good optical and electrical properties. Its refractive index value of n=1.85 at 620.25 nm can be cited as confirmation. Figure 1 shows the variation of the plasmonic structure, which refers to the possibility of varying the thickness of the metal and AZO films.

The experimental measurements related to this research activity are reported in [23].

**Figure 2:**
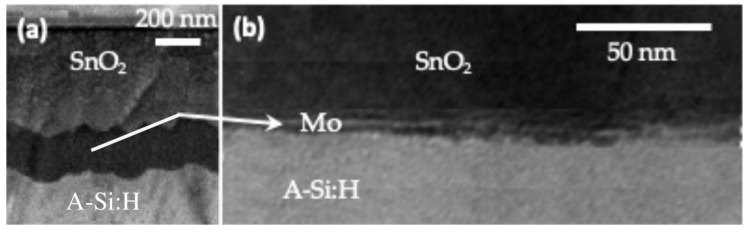
(**a**) Cross-section of a solar cell based on the structure of Figure 1 stack from SEM analysis (**b**) Cross-section of the cell stack from TEM analysis.

Figure 2 shows a cross-sectional scanning electron microscopy (SEM) micrograph of a cell based on the structure of Figure 1.

The values of the SPP decay length may vary depending on the material. For the dielectric layer, it is of the order of 100 nm in the visible range, while in a metal, it is close to the value of the metal skin depth at visible frequencies.

In this study, we used molibdenium (Mo) as metal because for Mo, we observed an absolute value of $\varepsilon_{m}$ of about 10 in units of the vacuum dielectric constants. This implies that in the case of Mo, the SPP modes at the metal amorphous/silicon (A-Si:H) interface are at wavelengths higher than ≈500 nm, respectively. This results in a longer SPP lateral propagation, and therefore the SPP waves can reach the side contacts of the structure without undergoing excessive attenuation [24,25,26,27,28].

Molibdenum has a specific dielectric constant value. For λ = 620.25 nm it is $\varepsilon_{Mo}=-72.983+13.377i$ [29]. The value consists of a real part, which in the case of Mo is negative, and an imaginary part, which is positive, but of low value. The dielectric/metal interface was defined as a perfectly matched layer (PML) in the FEM analysis. This is the boundary condition through which the artificial absorbing layer of the wave equations was introduced. The result of the FEM analysis showed a change in the value of the SPP energy on the two surfaces considered, which refers to the dependence of the SPP energy on the thickness of the active layer, as mentioned earlier.

### 2.3. Experiences in the Investigation of SPP and Simulation Results by COMSOL

In this paper, we investigate the main properties of SPP and their dispersion relation with respect to the variation in the thicknesses of a multilayer structure (glass/oxide/metal/dielectric) at fixed excitation frequency.

By solving the full wave 3D Maxwell equations in the simple geometry shown in Figure 1 using the finite element method-based software package COMSOL Multiphysics, we have obtained the $\lambda_{SPP}$ and $L_{SPP}$ data values for different thickness values. The perfectly matched layer boundary condition was chosen for the external surface of the plasmon structure. The exciting wave was monochromatic on the visible spectra and ranged from 400 nm to 700 nm. It should be noted that large-range excitation frequency led to a change in the value of the permittivity. The optimal thickness of aluminum-doped zinc oxide (AZO) led to a couple of values of $\lambda_{SPP}$ and $L_{SPP}$.

The geometry and the involved materials in a multilayer structure with rated monochromatic wave led to a value of the energy relative to the SPPs propagation, which changed with different pairs of thicknesses values. The feed-forward neural networks (FFNNs) provide the optimal propagation relative to a couple of thicknesses, which in turn leads to improved efficiency of the photovoltaic device. We have developed the nanoplasmonic structure in COMSOL RF Module, which incorporates effects of the electromagnetic waves. The developed model is a three-dimensional model in which the electromagnetic field is simulated. First, the magnetic field is analysed, and then the wavelength $\lambda_{SPP}$ is calculated by a Matlab script. The AZO thickness is fixed in this structure. Meanwhile, the metal thickness is modified in a wide range and then in the reverse. The main aim of the FEM-based simulations was to find a magnetic field for different structural dimensions at the interfaces between AZO/Metal and Metal/dielectric. The calculation of $\lambda_{SPP}$ was performed by Matlab software, creating a script.

The values of $\lambda_{SPP}$ and $L_{SPP}$ were computed for the visible range of the wavelength at the following different thickness values *t* of the metal: 36 nm, 42 nm, 48 nm, 54 nm, 60 nm, 72 nm, 84 nm, 96 nm, and 128 nm.

## 3. The Proposed CFNN-Based Architecture

Generally, the design of a multilayer plasmonic structure involves the design of finite elements method (FEM) models, which depend on a set of construction parameters of the physical solar cell. By means of such empirical models and several simulation runs, an optimal design of the physical cell could be derived. On the other hand, the calculation of $\lambda_{SPP}$ and $L_{SPP}$ carried out by means of the Equations (4) and (5) refers to infinitely extended ideal plasmonic structures in which there is no defect in the materials. In addition, in this calculation, the permittivity of materials is considered to be constant when compared with the frequency of the incident wave change.

**Figure 3:**
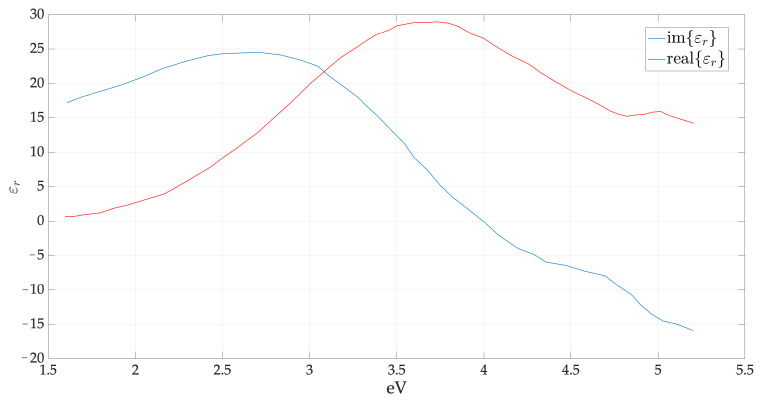
The real and imaginary part of amorphous silicon and doped relative dielectric constant as a function of photon energy.

Thus, in practical applications, the calculations carried out using Equations (4) and (5) are not in agreement with the experimental data. For the calculation of $\lambda_{SPP}$ and $L_{SPP}$ in real plasmonic structures, it is necessary to use the solution of Maxwell’s equations by means of the finite element method. However, FEM models are very computationally intensive, and such an approach is unfeasible when seeking the optimal construction parameters for an extended range. In addition, a wide range of exciting frequencies leads to a change in the value of the permittivity (see Figure 3). The geometry and the materials of a multilayer structure, with a rated monochromatic wave, affect the value of the energy relative to the SPPs propagation, which changes according to different pairs of thickness values. For this reason, we begin the investigation by means of the finite elements method (FEM) of the plasmonic phenomena in terms of propagation characteristics in a multilayer structure with an Aluminum-doped Zinc oxide (AZO). The latter is an indium-free TCO material for optoelectronics.

In this paper, we propose to overcome this problem by building a neural-network model. The outcome of the proposed approach is a drastic reduction in execution times (from hours with the FEM solution to seconds with neural network) for the model of a physical cell, which in turn makes it possible to find an optimal design for a plasmonic structure.

In this study, a Cascade Forward Neural Network (CFNN), which is a class of neural networks, was selected. The choice of CFNN is dictated by its properties. Compared to feed-forward networks, it includes, in addition to the linear connections of successive layers in the output direction that occur in both cases, connections from the input to each of the following layers. As a result, the use of CFNN carries some advantages, such as minimizing the prediction spread [30].

**Figure 4:**
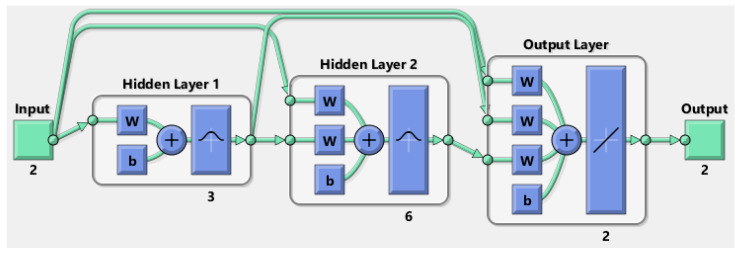
Architecture of the selected CFNN.

The proposed and implemented CFNN architecture is depicted in Figure 4. Three neurons with a radial basis transfer function are embedded in the first and second hidden layers, while the output layer is characterized by a linear transfer function. The relative learning parameters and the Levenberg–Marquardt algorithm (LMA) are used for the CFNN. It is important to avoid overfitting in the networks. For this reason, the early stopping rule is implemented [29,31,32,33].

The CFNN topology requires input data to be provided for testing. The more input data available, the more satisfactory the result will be. For this purpose, COMSOL Multiphysics software dedicated, among others, to the simulation of SPP characteristics was used. For the given boundary conditions, 3D Maxwell equations were solved, which allowed for multiple inputs in the CFNN study [34].

For both $\lambda_{SPP}$ and $L_{SPP}$, the error is lower than a realistic measurement a priori error. The average RMS for the predicted $\lambda_{SPP}$ is less than 7%, while the average RMS for $L_{SPP}$ is less than 6%.

## 4. Simulation Results

Various solar cell technology solutions are available on the market. Among the most widely used are silicon wafer devices. In the research, a different type of cells has been studied: thin film solar cells with thicknesses in the range of 1–2 μm [35,36]. They are classified as the second generation of photovoltaic devices. Currently, they are used much less than the first-generation cells (silicon wafers), but the demand for them has grown significantly over the past few years. Important factors affecting the energy value with respect to SPP propagation are the geometry of the multilayer structure and the material from which it is formed. Depending on the thickness of the individual layers, the energy values change.

The neural network considered in this study operates on the input signals, which here are the thicknesses of the active layers, namely AZO and metal, and the frequency of the exciting wave. Inputs are also provided by the FEM analysis, whereby input values of the electric field are delivered to the CFNN. This allows the dedicated neural network to obtain two values relating to the optimal propagation. In this way, the solar energy conversion efficiency can be increased.

The used CFNN-based approach provides a new and powerful way to discover the nonlinear mapping related to the parameters of the design process and the optimal outputs that mainly characterize the involved plasmonic phenomena at the interfaces AZO/METAL and METAL/DIELECTRIC.

A large number of simulations concerning the SPP propagation in the investigated multilayer plasmonic structure were performed by changing metal and AZO thicknesses in the range 28–40 nm for the metal and in the range 20–120 nm for the AZO for the two values of ε as $\varepsilon_{Mo}=-72.983+13.377i$, ε=−4.8+22.2i [26].

**Figure 5:**
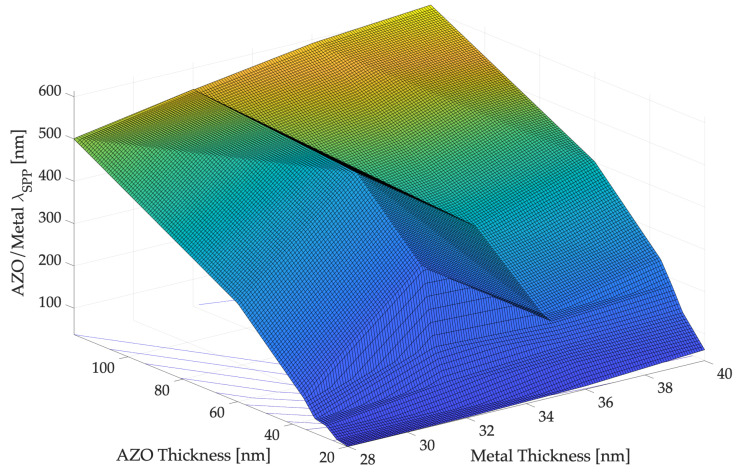

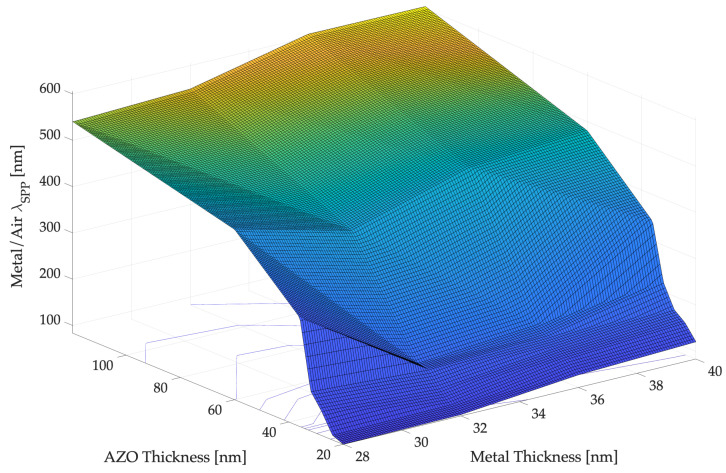
The plasmon’s wavelength, $\lambda_{SPP}$ for different thicknesses values of metal and AZO at the interface AZO/METAL (**up**) and METAL/DIELECTRIC (**down**) obtained by means of COMSOL simulations.

**Figure 6:**
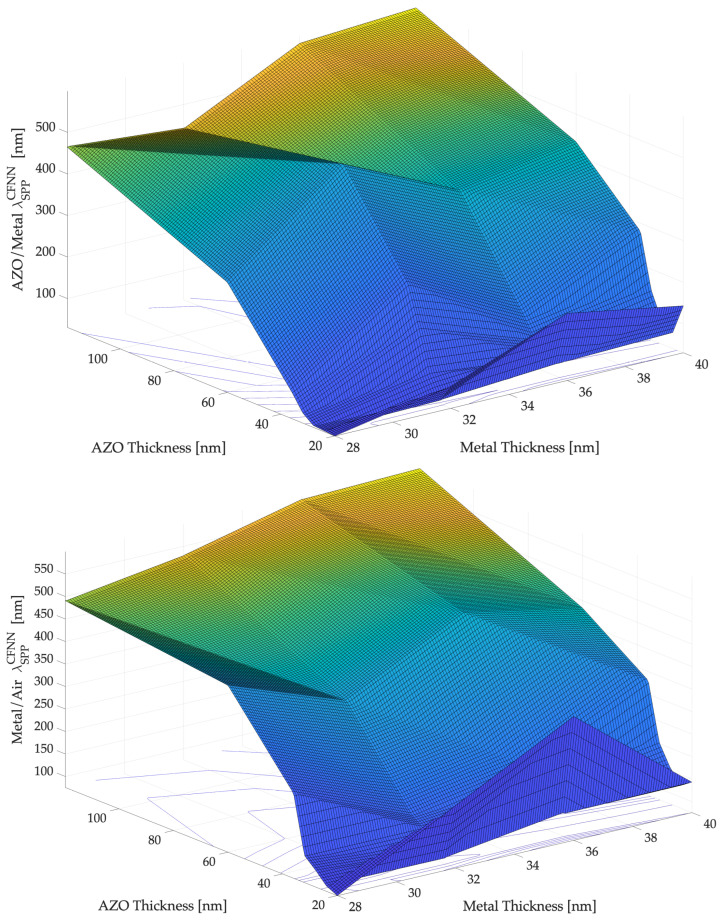
The plasmon’s wavelength, $\lambda_{SPP}$ for different thicknesses values of metal and AZO at the interface AZO/METAL (**up**) and METAL/DIELECTRIC (**down**) obtained by means the use of selected CFNN.

Figure 5 and Figure 6 show the simulation results, while the most significant results are summarized in Table 1, proving that the variations in material thicknesses affect the optimal plasmonic propagation values. The main simulations results are included, where $\lambda_{SPP-CFNN}$ is the neural value computed by the selected CFNN presented in the previous section expressed in nm.

Lately, a prototype of a photovoltaic cell based on the structure analyzed in the present study has been realized at the Institute for Microelectronics and Microsystems IMM of Catania, Italy.

The results obtained with respect to any non-plasmonic cell with similar characteristics are: an increase in light absorption at the plasmonic resonance frequencies of 500%, an increase in external quantum efficiency for the photovoltaic prototype of 20–30%, and an increase in the fill factor for the photovoltaic prototype of 5–10%.

## 5. Conclusions

It is essential that sensors possess characteristics such as high sensitivity and ease of manipulation under different conditions. For this reason, in order to improve the performance of the sensor, more complex cells are realized: with coatings, metallic back contacts, multi-junctions, etc. It is therefore of great importance to investigate the relationship between geometries and materials used for the realization of these devices and their propagation characteristics.

In this paper, a CFNN has been developed to achieve the inner relationships between the excitation wavelength of SPPs, AZO and metal thicknesses and $\lambda_{SPP}$ and $L_{SPP}$ considering the incident light for exciting SPPs. Furthermore, the optimal values of AZO and metal thicknesses were determined in the structure to improve the conversion efficiency for plasmonic devices. With these values so determined, we obtained a conversion efficiency increase of 12% in all realized devices.

The developed neural network architecture proved to be effective and efficient, as supported by the simulation results. The aim was to find out the optimal characteristics wavelengths of the SPPs to improve the propagation efficiency that, in turn, led to an enhancement of the energy conversion efficiency in solar cells. Additionally, a comparison with standard and different plasmonic materials, at several thicknesses values, revealed the promising capabilities of AZO as compact and low-loss plasmonic material.

Plasmonic structures made with crystalline silicon need more than 100 microns of material to absorb light, while those realized with the thin-film technology require only 1 micron. Considering that low-cost materials are used to make the latter, they have a lower cost of about 30%. On the other hand, poly and mono crystalline silicon plasmonic structures have a higher efficiency than thin-film ones: 22% of the former against 13% of the latter. When optimizing thicknesses by using the developed neural network architecture, it is possible to increase the efficiency of the plasmonic structures by up to 14–15%, thus making this technology competitive.

## Figures and Tables

**Table 1:** Simulation results of the SPP propagation by CFNN: $\lambda_{SPP-CFNN}$ by changing thickness values (expressed in nm).

METAL	AZO	AZO/	METAL/	AZO/	METAL/
		METAL	DIELECTRIC	METAL	DIELECTRIC
Thickness	Thickness	$\lambda_{SPP}$	$\lambda_{SPP}$	$\lambda_{SPP}^{CFNN}$	$\lambda_{SPP}^{CFNN}$
28	20	37.05	83.22	30.15	75.43
28	24	42.92	93.26	32.23	87.38
28	28	65.17	135.24	34.46	113.02
28	32	71.17	169.04	54.89	135.52
28	36	108.67	318.31	94.13	263.98
28	60	269.04	450.79	295.15	446.10
28	120	501.05	540.81	465.36	490.80
32	20	38.55	85.86	83.00	213.93
32	24	47.45	97.24	38.60	86.47
32	28	100.37	122.94	93.45	96.17
32	32	174.52	154.55	193.00	135.84
32	36	350.79	159.12	288.38	154.61
32	60	510.94	386.39	512.72	350.59
32	120	549.42	550.41	442.89	530.02
36	20	45.07	110.42	191.69	350.56
36	24	90.21	135.23	58.99	124.69
36	28	120.21	150.21	65.36	140.24
36	32	163.92	165.83	97.67	160.61
36	36	174.56	180.31	143.19	203.18
36	60	316.38	460.75	383.39	483.93
36	120	591.77	607.16	581.65	593.24
40	20	62.92	120.47	143.19	143.19
40	24	96.59	151.74	67.83	126.37
40	28	130.21	170.62	84.00	156.47
40	32	184.56	220.21	150.12	203.33
40	36	234.19	342.35	280.42	330.55
40	60	401.57	480.04	433.27	433.27
40	120	615.13	605.73	600.37	600.83

## Data Availability

Not applicable.

## References

[1] Fischer I.A., Augel L., Kropp T., Jitpakdeebodin S., Franz N., Oliveira F., Rolseth E., Maß T., Taubner T., Schulze J. (2016). Ge-on-Si PIN-photodetectors with Al nanoantennas: The effect of nanoantenna size on light scattering into waveguide modes. Appl. Phys. Lett..

[2] Schlipf J., Fischer I.A. (2021). Rigorous coupled-wave analysis of a multi-layered plasmonic integrated refractive index sensor. Opt. Express.

[3] Badshah M.A., Koh N.Y., Zia A.W., Abbas N., Zahra Z., Saleem M.W. (2020). Recent developments in plasmonic nanostructures for metal enhanced fluorescence-based biosensing. Nanomaterials.

[4] Alkhalayfeh M.A., Aziz A.A., Pakhuruddin M.Z. (2021). An overview of enhanced polymer solar cells with embedded plasmonic nanoparticles. Renew. Sustain. Energy Rev..

[5] Xu K.D., Guo Y.J., Yang Q., Zhang Y.L., Deng X., Zhang A., Chen Q. (2021). On-chip GaAs-based spoof surface plasmon polaritons at millimeter-wave regime. IEEE Photonics Technol. Lett..

[6] ElKhamisy K., Abdelhamid H., Elagooz S., El-Rabaie E.S. (2021). The effect of different surface plasmon polariton shapes on thin-film solar cell efficiency. J. Comput. Electron..

[7] Li G., Li H., Ho J., Wong M., Kwok H.S. (2015). Ultra-thin, high performance crystalline silicon tandem cells fabricated on a glass substrate. Sol. Energy Mater. Sol. Cells.

[8] D’Agata R., Spoto G. (2012). Surface Plasmon Resonance-Based Methods. Detection of Non-Amplified Genomic DNA.

[9] Atwater H.A., Polman A. (2010). Plasmonics for improved photovoltaic devices. Nat. Mater..

[10] Guo C., Wang J., Chen X., Li Y., Wu L., Zhang J., Tao C.A. (2018). Construction of a biosensor based on a combination of cytochrome c, graphene, and gold nanoparticles. Sensors.

[11] Yusoff S.F.A.Z., Lim C.S., Azzuhri S.R., Ahmad H., Zakaria R. (2018). Studies of Ag/TiO_2_ plasmonics structures integrated in side polished optical fiber used as humidity sensor. Results Phys..

[12] Butt M., Khonina S., Kazanskiy N. (2021). Plasmonics: A necessity in the field of sensing-a review. Fiber Integr. Opt..

[13] Badri S.H., Gilarlue M. (2020). Coupling between silicon waveguide and metal-dielectric-metal plasmonic waveguide with lens-funnel structure. Plasmonics.

[14] Cai M., Wang S., Gao B., Wang Y., Han T., Liu H. (2018). A new electro-optical switch modulator based on the surface plasmon polaritons of graphene in mid-infrared band. Sensors.

[15] Devasia S., Athma P., Raphael R., Anila E. (2022). Effect of source-substrate distance on the transparent electrode properties of spray pyrolysed aluminium doped zinc oxide thin films. Mater. Today Proc..

[16] Kumar P., Dharmaprakash S., Patil P.S., Neelamma B. (2021). Ellipsometric and third-order nonlinear optical studies of pulsed laser deposited aluminium doped zinc oxide thin films. Mater. Today Proc..

[17] Maitra S., Pal S., Datta S., Maitra T., Dutta B., Roy S. (2021). Nickel doped molybdenum oxide thin film counter electrodes as a low-cost replacement for platinum in dye sensitized solar cells. Mater. Today Proc..

[18] Baek S., Kim J., Choo S., Sen A., Jang B., Pujar P., Kim S., Kwon H.J. (2022). Low-Temperature Carrier Transport Mechanism of Wafer-Scale Grown Polycrystalline Molybdenum Disulfide Thin-Film Transistor Based on Radio Frequency Sputtering and Sulfurization. Adv. Mater. Interfaces.

[19] Khashan K.S., Sulaiman G.M., Hussain S.A., Marzoog T.R., Jabir M.S. (2020). Synthesis, characterization and evaluation of anti-bacterial, anti-parasitic and anti-cancer activities of aluminum-doped zinc oxide nanoparticles. J. Inorg. Organomet. Polym. Mater..

[20] Zhao X., Shen H., Zhang Y., Li X., Zhao X., Tai M., Li J., Li J., Li X., Lin H. (2016). Aluminum-Doped Zinc Oxide as Highly Stable Electron Collection Layer for Perovskite Solar Cells. ACS Appl. Mater. Interfaces.

[21] Zhang P., Hong R., Chen Q., Feng W. (2014). On the electrical conductivity and photocatalytic activity of aluminum-doped zinc oxide. Powder Technol..

[22] Li H., Hu Y., Yang Y., Zhu Y. (2020). Theoretical investigation of broadband absorption enhancement in a-Si thin-film solar cell with nanoparticles. Sol. Energy Mater. Sol. Cells.

[23] Lombardo S., Battaglia A., Foti M., Tringali C., Cannella G., Costa N., Gerardi C., Principato F. (2014). Plasmonic modes in molybdenum ultra-thin films suitable for hydrogenated amorphous silicon thin film solar cells. Energy Procedia.

[24] Fujiwara H., Kondo M. (2007). Effects of a-Si: H layer thicknesses on the performance of a-Si: H/c-Si heterojunction solar cells. J. Appl. Phys..

[25] Murthy M., Tembhurne S., Ganguly S. (2012). Co-optimizing plasmonic and solar cell structures. Proceedings of the 2012 12th IEEE International Conference on Nanotechnology (IEEE-NANO).

[26] Lombardo S., Tringali C., Cannella G., Battaglia A., Foti M., Costa N., Principato F., Gerardi C. (2012). Plasmonic effects of ultra-thin Mo films on hydrogenated amorphous Si photovoltaic cells. Appl. Phys. Lett..

[27] Yuan Y., Ding L., Guo Z. (2011). Numerical investigation for SPR-based optical fiber sensor. Sens. Actuators B Chem..

[28] Moznuzzaman M., Islam M.R., Hossain M.B., Mehedi I.M. (2020). Modeling of highly improved SPR sensor for formalin detection. Results Phys..

[29] Capizzi G., Bonanno F., Napoli C. (2011). Hybrid neural networks architectures for SOC and voltage prediction of new generation batteries storage. Proceedings of the 2011 International Conference on Clean Electrical Power (ICCEP).

[30] Bonanno F., Capizzi G., Gagliano A., Napoli C. (2012). Optimal management of various renewable energy sources by a new forecasting method. Proceedings of the Symposium on Power Electronics, Electrical Drives, Automation and Motion (SPEEDAM).

[31] Bonanno F., Capizzi G., Napoli C. (2012). Some remarks on the application of rnn and prnn for the charge-discharge simulation of advanced lithium-ions battery energy storage. Proceedings of the Symposium on Power Electronics, Electrical Drives, Automation and Motion (SPEEDAM).

[32] Capizzi G., Bonanno F., Tina G.M. (2011). Recurrent neural network-based modeling and simulation of lead-acid batteries charge–discharge. IEEE Trans. Energy Convers..

[33] Capizzi G., Bonanno F., Napoli C. (2011). Recurrent neural network-based control strategy for battery energy storage in generation systems with intermittent renewable energy sources. Proceedings of the 2011 International Conference on Clean Electrical Power (ICCEP).

[34] Nam W.J., Ji L., Varadan V.V., Fonash S.J. (2011). Designing optical path length, photonic, and plasmonic effects into nanostructured solar cells. Proceedings of the 2011 11th IEEE International Conference on Nanotechnology.

[35] Catchpole K., Polman A. (2008). Design principles for particle plasmon enhanced solar cells. Appl. Phys. Lett..

[36] Bai W., Gan Q., Song G., Bartoli F. (2010). Plasmonic back structures designed for efficiency enhancement of thin film solar cells. Proceedings of the Conference on Lasers and Electro-Optics (CLEO) and Quantum Electronics and Laser Science (QELS).

